# Initiation of a novel text messaging system in total knee and hip arthroplasty

**DOI:** 10.1186/s42836-024-00265-z

**Published:** 2024-08-04

**Authors:** Johannes M. van der Merwe, Michaela E. Nickol

**Affiliations:** Adult Reconstruction Subdivision, Orthopaedic Division, University of Saskatchewan, Saskatoon, SK S7K0M5 Canada

**Keywords:** Text messages, Joint arthroplasty, Patient satisfaction, Emergency, Phone calls

## Abstract

**Background:**

The primary objective of this study was to investigate whether using a novel text messaging system improves patient overall satisfaction compared to standard care. Secondary objectives included assessing the impact of the text messaging system on decreasing narcotic usage, the number of emergency department visits, the range of flexion and extension, and number of telephone calls to the surgeon’s office.

**Methods:**

We enrolled 217 patients to either receive informative text messages (text messaging group, *n* = 86) or no additional text messages (conventional group, *n* = 131). Patients self-reported results on a questionnaire at the 6-week follow-up regarding the primary and secondary objectives. The active range of motion of total knee arthroplasty patients was recorded by the surgeon or treating physiotherapist.

**Results:**

There was no significant difference in overall satisfaction (*P* = 0.644), narcotic cessation (*P* = 0.185), range of motion (Flexion *P* = 0.521; Extension *P* = 0.515), and emergency department visits (*P* = 0.650) between the two groups. There was a statistically significant decrease in surgeon office calls favoring the text messaging group (*P* = 0.029). A subgroup analysis revealed that the statistical difference was mainly in the TKA group (*P* = 0.046).

**Conclusions:**

A novel text messaging system may help reduce the work burden by decreasing telephone calls to the surgeon’s office. While satisfaction, narcotic usage, emergency department visits, and range of motion did not significantly differ, patients endorsed the system for friends/family.

**Supplementary Information:**

The online version contains supplementary material available at 10.1186/s42836-024-00265-z.

## Introduction

The demand for total knee (TKA) and hip (THA) arthroplasty has been on the rise due to the aging population [1]. Even though arthroplasties are successful surgeries [2], surgeons are continually trying to improve outcomes. To achieve better surgical outcomes, the Enhanced Recovery After Surgery Society (ERAS) proposed that perioperative education, among many other modalities, should play an important role in improving surgical outcomes [3]. A great many ways are available to deliver information to patients in a timely fashion. The standard platform currently in place at our institution utilizes booklets, pamphlets, instructional videos, and perioperative private or group sessions. Occasionally, this can be quite challenging for rural patients who cannot make multiple trips to the city for the sessions. While booklets and pamphlets describe the exercises needed, they are not as instructional as videos [4, 5]. With new technologies available to us (e.g., smartphones), informative texts and videos can be delivered to patients instantly and in the convenience of their homes. Understandably, there will be a small group of patients who do not own a smartphone, tablet, or computer or simply do not want to participate. However, realistically, most patients would be able to take advantage of the technologies to enhance their care. Text messages deliver timely information, could identify postoperative problems early, and offer resolutions. It also might increase patient compliance with exercise regimes, and lead to early discontinuation of narcotics due to their automation capabilities [6]. Companies offering text messaging services mention additional hypothetical benefits that include increased satisfaction, decreased calls to the surgeon’s office, decreased opiate usage, decreased emergency department visits, and the ability to support the patient outside of the traditional clinical setting [7].

We conducted this study to assess if initiating a novel text messaging service at our institution will improve the satisfaction rates in patients undergoing total hip and knee arthroplasty compared to the conventional group. In addition, we assessed if a novel text messaging service could decrease calls to the surgeon’s office and decrease emergency department visits. We calculated if patients’ knee range of motion would improve with receiving text messages and exercise videos, and if patients stopped their narcotic usage earlier compared to the group that did not receive text messages.

## Patients and methods

**Fig. 1:**
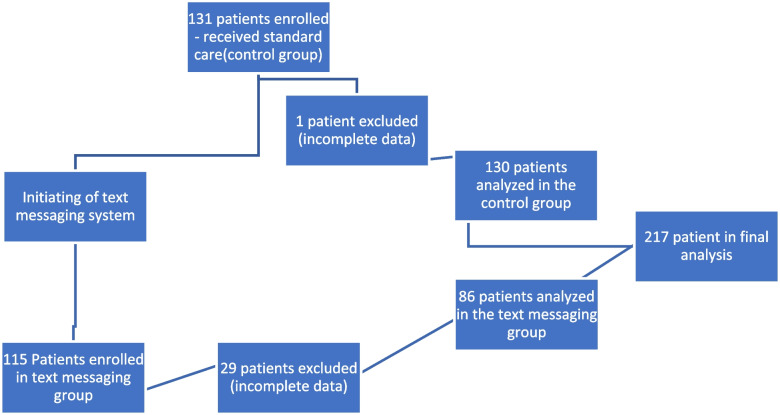
Outline of the enrollment

After obtaining institutional ethics approval (Bio-REB 4078), the novel text messaging system was implemented on the 1st of April, 2023. All patients were given instructions concerning how to enroll in the text messaging system via an information sheet from the text messaging company mailed to them 1–2 months prior to surgery, in addition to the standard of care, which included booklets supplied to them by the health region. The information sheet explained the purpose of the novel text messaging system and gave patients instructions on how to “join” on their smartphones. Patients were excluded from the analysis if they did not have a smartphone, did not have access to a smartphone through a friend or family member, or did not want to enroll. If patients did not “join” before surgery, another discussion between the surgeon and the patient occurred prior to surgery. The purpose of the text messaging system was explained, and patients were given the opportunity to participate if they chose. Patients were informed that they could “opt out” of the text messaging system at any time if they did not find it useful. Since implementation, we have enrolled 115 patients to receive text messages (See Fig. 1). Twenty-nine patients who underwent a total hip-or-knee arthroplasty, were excluded due to incomplete data collection or not enrolling due to lack of a smartphone or unwillingness to partake. Text messages commenced 2 weeks before surgery and continued for 6 weeks following surgery. Patients would also receive a reminder one year post-surgery to make an appointment for their annual follow-up. THA and TKA patients received a total of 150 and 159 messages over a 8-week period respectively (See Supplementary Material A). The messages were overall similar for all the patients, with unique differences between the TKA and THA groups. The individual text messages varied, ranging from how to prepare for surgery (i.e., which personal belongings to bring on the day of their surgery, when to prescribe, and preoperative exercises) to postoperative exercises, pain management strategies postoperatively when to make appointments to see their family physician or surgeon and when to wean of narcotics. In addition, patients also received encouraging messages to help them through the recovery period. For the most part, they were informative texts without an option to reply. Periodically, there is an opportunity for patients to interact with the text messaging system. An example would be a text message encouraging patients to start weaning of their narcotics. The message would state that if a patient would like help with the weaning process, to respond to the text with the word “wean”. Subsequent texts will then follow, giving the patient information to wean of their narcotics safely. Unfortunately, there is a cost associated with the use of the text messaging system but anybody can afford it.

Before initiating the novel text messaging system in April 2023, we enrolled 131 patients (conventional group). These patients only received a booklet, per our health region’s standard of care. The booklet describes perioperative exercises, types of surgical procedures performed, and realistic expectations following surgery. All the surgeries occurred at a tertiary high-volume center. A single high-volume adult reconstruction surgeon performed all surgeries. Total knee arthroplasties were performed via a medial parapatellar approach. Patella resurfacing was performed selectively, depending on the severity of patella osteoarthritis or a history of inflammatory conditions. A medial congruent total knee replacement (Persona, Zimmer Biomet, Warsaw, In, USA) was utilized in all cases. A periarticular block was used in all cases. Adductor canal block was employed selectively in patients *as per* anesthesiologist’s recommendations. Postoperatively, patients were fully weight-bearing with no restrictions.

All total hip arthroplasties were performed via a posterolateral approach. A large femoral head (≥ 36) was utilized in all cases. Patients were fully weight-bearing postoperatively in all cases and were instructed to exercise limited hip precautions for 12 weeks following surgery.

Patients were instructed to contact the surgeon’s office for any questions they might have in the postoperative period. Patients routinely followed up with their general practitioner at 2 weeks for a wound check and at 6 weeks with their surgeon. At the six-week mark, questionnaires were distributed to patients by the receptionist. The self-reported questionnaires enquired about when patients discontinued narcotics usage, how many times they presented to the emergency department and the number of calls a patient made to the surgeon’s office (See Supplementary Material A). In addition, patients were asked to rate their overall satisfaction on a scale ranging from 0 (worse) to 10 (exceptional). The operating surgeon or treating physiotherapist performed active total knee arthroplasty measurements via a goniometer and documented the results. All patients had access to office support during working hours if required. The emergency department dealt with emergencies that occurred after hours or in the evenings.

### Statistical analysis

A comparative analysis of demographic characteristics among the investigated cohorts was undertaken to identify potential statistically significant disparities in group composition. Depending on the nature of the variables under comparison, either Fisher’s exact test or Student’s *t*-test was employed. The results of the implementation of the new text messaging system across the examined groups were compared using Fisher’s exact test or Mann–Whitney U test, selected in accordance with the data type. All the selected methodologies represented established instruments for detecting pertinent distinctions between groups. Significance level was determined as *P* < 0.05. The statistical power for all the variables was calculated and determined to be 0.9 or higher, with the accepted standard being 0.8 or higher.

## Results

We enrolled 246 patients and only 217 patients were analyzed due to incomplete data in 30 patients. When comparing the text messaging and conventional groups we found no statistically significant differences in their BMI (*P*-value 0.054), sex (*P*-value 0.087), or age (*P*-value 0.696). The text messaging group had 68 total knee arthroplasties (TKA) and 20 total hip arthroplasties (THA). The conventional group had 71 TKA’s and 47 THA’s. The differences were statistically significant (*P*-value 0.011) (see Table 1).

**Table 1:** Demographic data between the conventional group and the text messaging group. SD = standard deviation. A statistical difference was seen in the proportion of TKA and THA in the text messaging and control group (*P* = 0.011)

	**Received messages** **Mean ± SD**	**Did not receive messages** **Mean ± SD**	***P*-value**
Sex	79 men, 36 women	76 men, 55 women	0.087
Age	67.93 ± 11.217	67.43 ± 8.451	0.696
Height	166.81 ± 10.36	167.84 ± 9.97	0.690
Weight	93.54 ± 22.22	90.32 ± 19.4	0.227
BMI	33.85 ± 8.73	32 ± 6.08	0.054
TKA + THA	TKA 68, THA 20	TKA 71, THA 48	**0.011**

**Fig. 2:**
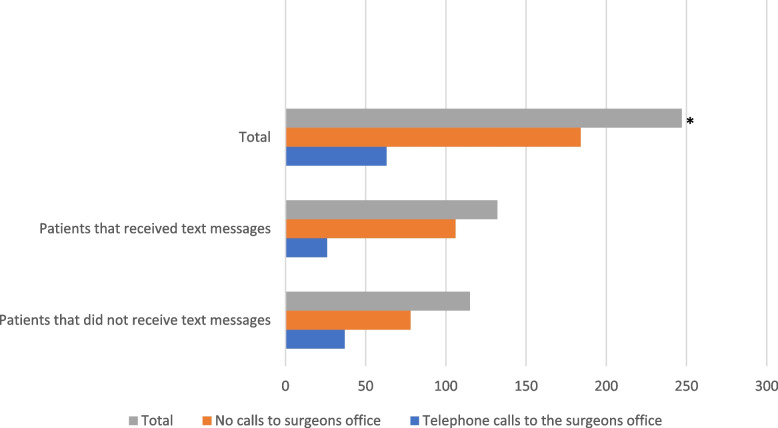
Calls made by both TKA and THA patients to the surgeon’s office. *P = 0.0285

Comparing the two groups, we found no statistically significant difference between them in the duration of narcotic usage (*P* = 0.185), emergency department visits (*P* = 0.650), and satisfaction rates (*P* = 0.644). Looking at only the TKA patients in both groups, we found no difference in flexion (*P* = 0.521), and extension (*P* = 0.515). Interestingly, the telephone calls to the surgeon’s office were significantly less in the text messaging group compared to its conventional counterpart (*P* = 0.029) (Fig. 2; Tables 2 and 3).

**Table 2:** All patients (TKA and THA) made fewer phone calls to the surgeon’s office that received text messages, *P* = 0.0285

	**Telephone Calls to Surgeons Office**		**Total**
	**Yes**	**No**	
**Did patients (TKA + THA) receive text messages?**			
**Yes**	26	105	131
**No**	37	78	115
**Total**	63	183	246

**Table 3:** Clinical outcomes in the two group

	**Received Text** **Messages**	**Did Not Receive** **Text Messages**	***P*-value**
	**Mean ± SD**		
**Last Narcotic Dose**	12.47 ± 11.29	9.74 ± 8.212	0.185
**Satisfaction (rated from 0 to 10)**	9.41 ± 1.19	9.35 ± 1.345	0.644
	**Percentage**		
**Telephone Calls to Surgeons Office**	19.7% (yes); 80.3% (no)	32.2% (yes); 67.8% (no)	0.029
**Emergency Department Visits**	7.6% (yes); 94.2% (no)	9.6% (yes); 90.4% (no)	0.650

**Fig. 3:**
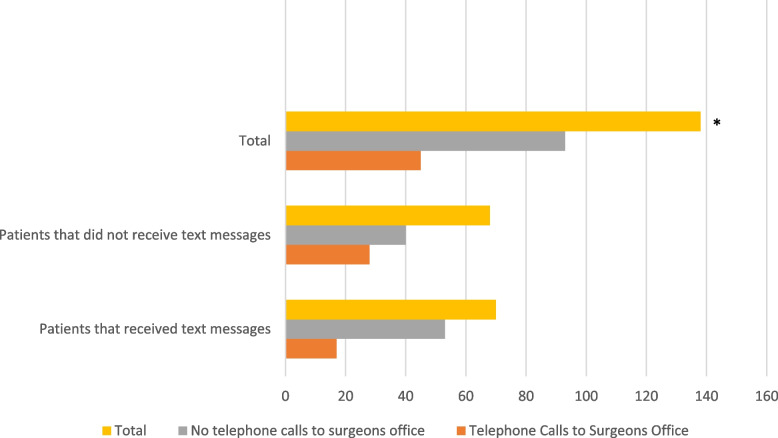
TKA patients’ calls to the surgeons office. *P = 0.0456

Our subgroup analysis revealed that there was no difference in narcotic duration (TKA: *P* = 0.174; THA: *P* = 0.183), emergency department visits (TKA: *P* = 0.778; THA: *P* = 1.000) and satisfaction rates (TKA: *P* = 0.779; THA: *P* = 0.308). Only the TKA group demonstrated a significantly reduced telephone calls to the surgeon’s office (TKA: *P* = 0.046; THA: *P* = 1.00) (See Table 4, Fig. 3).

**Table 4:** TKA patients made fewer calls to the surgeon’s office, *P* = 0.0456

	**Telephone Calls to Surgeons Office**		**Total**
	**Yes**	**No**	
**Did patients (TKA) receive text messages?**			
**Yes**	17	53	70
**No**	28	40	68
**Total**	45	93	138

In patients who subscribed to the text messages before surgery and those who only subscribed at the time of surgery, we found no difference in sex (*P* = 0.295), age (*P* = 0.279), BMI (*P* = 0.880), ratio between TKA and THA (*P* = 0.829), satisfaction rates (*P* = 0.264), flexion (only TKA) (0.237), extension (only TKA) (0.495), duration of narcotic usage (*P* = 0.985), emergency visits (*P* = 0.743) and telephone calls to the surgeon’s office(*P* = 0.294) (Table 5).

**Table 5:** Demographic data of the patients who received text messages prior to surgery and those who only received messages starting on the day of surgery

	**Received messages 2 weeks prior to surgery** **Mean ± SD**	**Only started receiving messages at time of surgery** **Mean ± SD**	***P*-value**
Sex	31 men, 10 women	47 men, 25 women	0.295
Age	67.29 ± 8.233	69.08 ± 8.447	0.279
Height	167.16 ± 11.2	166.07 ± 7.59	0.579
Weight	93.89 ± 21.77	93.7 ± 23.4	0.965
BMI	33.88 ± 8.71	34.15 ± 8.94	0.880
TKA + THA	TKA 22, THA 8	TKA 44, THA 12	0.829

There was no difference in likelihood of patients opting out of the text messaging system between the group that received text messages before surgery and the group that only subscribed at the time of surgery (*P* = 0.284). Both groups found text messaging useful (*P* = 0.532), and all the patients would recommend it to a friend or a family member (Table 6).

**Table 6:** People who didn’t opt out of the study found the text messages often, statistically more useful

	**Did you find the texts useful?**		**Total**
	**Yes**	**No**	
**Did you opt out of receiving text messages prior to 6 weeks?**			
**Yes**	7	0	7
**No**	106	0	106
**Total**	111	0	113

When we looked at the groups of patients that opted out of the text messaging system before 6 weeks postoperatively and the group that did not opt out, we found no difference in the duration of narcotics (*P* = 0.469), telephone calls to the surgeon’s office (*P* = 0.294), emergency department visits (*P* = 0.389), satisfaction rates (*P* = 0.302), extension (TKA patients) (*P* = 0.402), and flexion (TKA patients) (*P* = 0.702). Patients who did not opt out of the text messaging system prior to their 6-week follow-up, often found the texts useful (*P* = 0.027). Interestingly, both groups would recommend it to a family member or a friend (Table 6).

Seven patients opted out of receiving text messages prior to the end of the 6-week follow-up after surgery. Five of the seven patients had an ipsilateral joint replacement in the preceding 6 months. One patient decided to discontinue the text messaging system due to an inability to read the small text messages on their smartphone, while the other patient did not give a reason for discontinuation.

## Discussion

The volume of total hip and knee arthroplasties is expected to increase in the next decade [6]. With this mounting volume, calls to the surgeon’s office and emergency department visits are also expected to increase. In addition, Shah et al. [8] demonstrated an increase in work burden posed on the surgeon and their office personnel, with rapid pathway total joint arthroplasty. They found that, on average 2.5, telephone encounters taking place in the 1st week after surgery lasted over 11 min per patient. A novel automated text messaging system can deal with minor concerns that patients might have during their recovery, potentially decreasing the increased demand. Furthermore, the text messaging system also encourages patients to exercise, decreases and stops their narcotic usage earlier, and provides valuable information about realistic expectations, follow-up appointments, and pain-managing strategies.

In this study, a statistically significant difference was found between the two groups, with more total hip arthroplasties in the conventional group than in the text messaging group. This certainly can influence the outcome parameters, seeing that total hip arthroplasty and total knee arthroplasty usually follow a different postoperative course. Multiple studies demonstrated better postoperative outcomes following THA compared to TKA [9–11].

However, Judge et al. concluded that improvement after a TKA or THA was more commonly associated with preoperative expectations [12]. They found that patients with greater preoperative expectations were more likely to improve postoperative expectations. They determined that younger patients, women, increased body mass index (BMI), and higher education level were associated with superior preoperative expectations. In our study, even though there was a difference in number between total hip and knee arthroplasties, there were no statistically significant differences in age, sex or BMI. However, having more THAs in the conventional group could skew the satisfaction results in favour of the aforementioned group. Even with a further subgroup analysis looking at THA and TKA separately, we did not find a statistically significant difference in overall satisfaction rates between the conventional and text messaging groups.

In this study, telephone calls to the surgeon’s office were significantly reduced in the text messaging group. The subgroup analysis looking at TKA and THA separately revealed that only the TKA group showed a reduction in telephone calls to the surgeon’s office. This might be because TKA are generally more painful and symptoms typically tend to persist longer than THA [13]. In our study, we found that postoperative pain was the most frequent reason for calls to the surgeon’s office in both groups. Calls to the surgeon’s office were not affected by patients initiating the text messages preoperatively or doing it at the time of surgery. This makes inherent sense as most questions will be raised following surgery. There was no difference in the calls between patients continuing until the 6-week mark postoperatively and patients opting out of the text messaging system before their 6-week follow-up. The group of patients opting out of the text messaging system was under-powered, which should caution the readers of the validity of these findings. A study by Campbell et al. yielded similar findings, and they found that patients receiving text messages made, on average, 2 fewer calls to their surgeon’s office (*P* < 0.001) [6]. With improved education and information, it is reasonable to believe that fewer calls will be made to the surgeon’s office if patients are well-informed about what is normal and expected. This will free the surgeon and their office to deal with more urgent matters.

There has been a significant push to limit opioid prescriptions due to the opioid epidemic in the USA and orthopedic surgeons were reportedly the most frequent prescribers [13–17]. Perioperative text messaging is an effective tool for communicating with patients and therefore can be useful in decreasing opioid consumption [14, 18]. Campbell et al. demonstrated a statistically significant reduction in narcotic usage following TKAs and THAs [6]. They demonstrated that patients who used the text messaging system stopped their narcotics at a mean of 10 days earlier than the conventional group (*P* < 0.001) [6]. In our study, there existed no statistically difference in the duration of narcotic use between the groups or subgroups. We postulate that due to our current practice of prescribing limited amounts of narcotics for pain control, we are already limiting the amount of narcotics patients use postoperatively. Upon discharge, patients have been instructed to use alternative modalities (i.e., Tylenol, anti-inflammatories, icing) more frequently for pain management and only use the narcotics for breakthrough pain. This might explain why we could not observe a difference between the groups.

We also assessed the narcotic usage in patients who opted out of the text messaging system before their 6-week follow-up and did not observe a difference. This can be explained by the fact that five out of the seven patients had an arthroplasty performed within 6 months of the surgery, and, therefore, they might be well-versed in managing pain. In addition, the number of patients who opted out was small and not adequately powered to reach any definitive conclusions.

With the ever-increasing arthroplasty load and potential emergency department visits that might occur postoperatively, it is imperative for surgeons to ensure that only urgent issues should end up in the emergency department (ED) [19–21]. An automated text messaging system can deliver messages and information to patients to inform them about urgent issues that need to be addressed in the ED, or common findings following a total hip or knee arthroplasty that do not require and an ED visit. There are conflicting results about if an automated text messaging system might lead to a reduction of ED visits. One study found a reduction in ED visits with patients receiving text messages [6], while other studies did not see any difference [1, 7]. We did not find a difference in ED visits between the groups. Similarly, no difference was observed when we looked only at TKA or THA separately.

We failed to demonstrate an increase in satisfaction rates in the treatment group compared to the conventional group. This could be because of the relatively low patient numbers in both groups and the fact that both surgeries are already very successful. Song et al. had similar findings [2]. Their study may have been under-powered, and, therefore, might not be a true reflection of satisfaction rates associated with the introduction of text messages. Larger and more extensive studies are needed to assess if text messages positively impact satisfaction. Even though we could not demonstrate improved satisfaction statistically, clinically, patients did seem to value the text messaging system. It might also be worthwhile to explore patient-centered research to assess the impact text messaging might have on patients and experiences they lived.

Campbell et al. and others did demonstrate improved flexion and extension in the short-term follow-up (3 weeks) but failed to show the same improvement at 6 weeks [2, 6]. Similarly, in the TKA groups, we did not see a difference in the range of motion between the treatment and conventional groups at the 6-week mark. There was also no difference when patients received text messages 2 weeks prior to surgery, or when patients opted out of the text messages before the 6-week follow-up after surgery.

Xu et al. compared patients in a randomized controlled trial to look at the difference in outcomes between patients receiving a supervised physiotherapy program and patients doing a home-based enhanced knee-based flexion exercise. They did not find a difference between the groups at 1 year in the range of motion, pain scores, WOMAC AND KSS scores [22]. This echoed the aforementioned findings that patients might still get the same outcomes in the short term by doing the exercises *as per* text messages even if they miss structured, supervised physiotherapy sessions.

Our study does have some limitations. Firstly, the current study had a low external validity. All patients were operated on in a tertiary center by an adult reconstruction surgeon. However, any orthopedic surgeon can safely implement a text messaging system in any center. The text messaging system does not rely on surgeon qualifications or center amenities. Therefore, the results can be useful and applicable to surgeons interested in improving patient education and mitigating office burden.

Secondly, this was a non-randomized trial, which could mean that results obtained from this study might be due to the differences between the two groups rather than the intervention. We performed a subgroup analysis to address this limitation. The subgroup analysis results did not significantly alter the original groups. Unfortunately, in the subgroup analysis, the number of patients in each group became smaller, potentially leading to under-powered statistics. Investigators might fail to detect true important underlying differences in treatment effects across subgroups. Future properly-organized randomized controlled trials might help determine the efficacy of the text messaging system. Thirdly, we only enquired about emergency department visits. Certainly, there might be a small percentage of patients that presented to their family physicians’ office during after-hours time and the visits could be missed on the self-reported questionnaires. This could lead to under-reporting of the physician visits. Lastly, one potential limitation of our study was the presence of recall bias. As participants were asked to remember past events or behaviors, there was a possibility of inaccuracies or inconsistencies in their recollection, which could impact the validity of our findings. Future research incorporating more objective measures or longitudinal designs may help mitigate this limitation. Even though the difference in patient satisfaction between the text messaging group and the conventional group did not reach a statistically significant level, most patients found the text messaging favorable and would recommend it to their family or friends. This can be considered clinically meaningful.

## Conclusion

Instituting a novel text messaging system may help reduce the work burden by decreasing telephone calls to the surgeon’s office. Surgeons and their office personnel should understand that there might be an initial surge in workload with the introduction of the text messaging system, followed by a subsequent decrease in responsibility load. Even though there was not a statistically significant difference in satisfaction rates, duration of narcotic usage, or range of motion between the intervention and control group, an overwhelming majority of the patients found it helpful and all patients would recommend it to friends or family.

### Supplementary Information

Supplementary Material 1. Example of texts messages received by patients.

Supplementary Material 2. Patient satisfaction questionnaire.

## Data Availability

All the raw data and materials described in the manuscript are available, upon requests, to any scientist wishing to use them for non-commercial purposes.
