# Supplementary material for: Initiation of a novel text messaging system in total knee and hip arthroplasty
Source: Arthroplasty. 2024 Aug 4;6:43. doi: 10.1186/s42836-024-00265-z (PMC11298075; doi:10.1186/s42836-024-00265-z)
Supplement: Supplementary file 1 — Supplementary Material 1. Example of texts messages received by patients. [file 42836_2024_265_MOESM1_ESM.docx]

*Supplementary Material A: Patient satisfaction questionnaire.*
