# Supplementary figures and images for: Initiation of a novel text messaging system in total knee and hip arthroplasty
Source: Arthroplasty. 2024 Aug 4;6:43. doi: 10.1186/s42836-024-00265-z (PMC11298075; doi:10.1186/s42836-024-00265-z)

*Supplementary Material B: Example of texts messages received by patients.
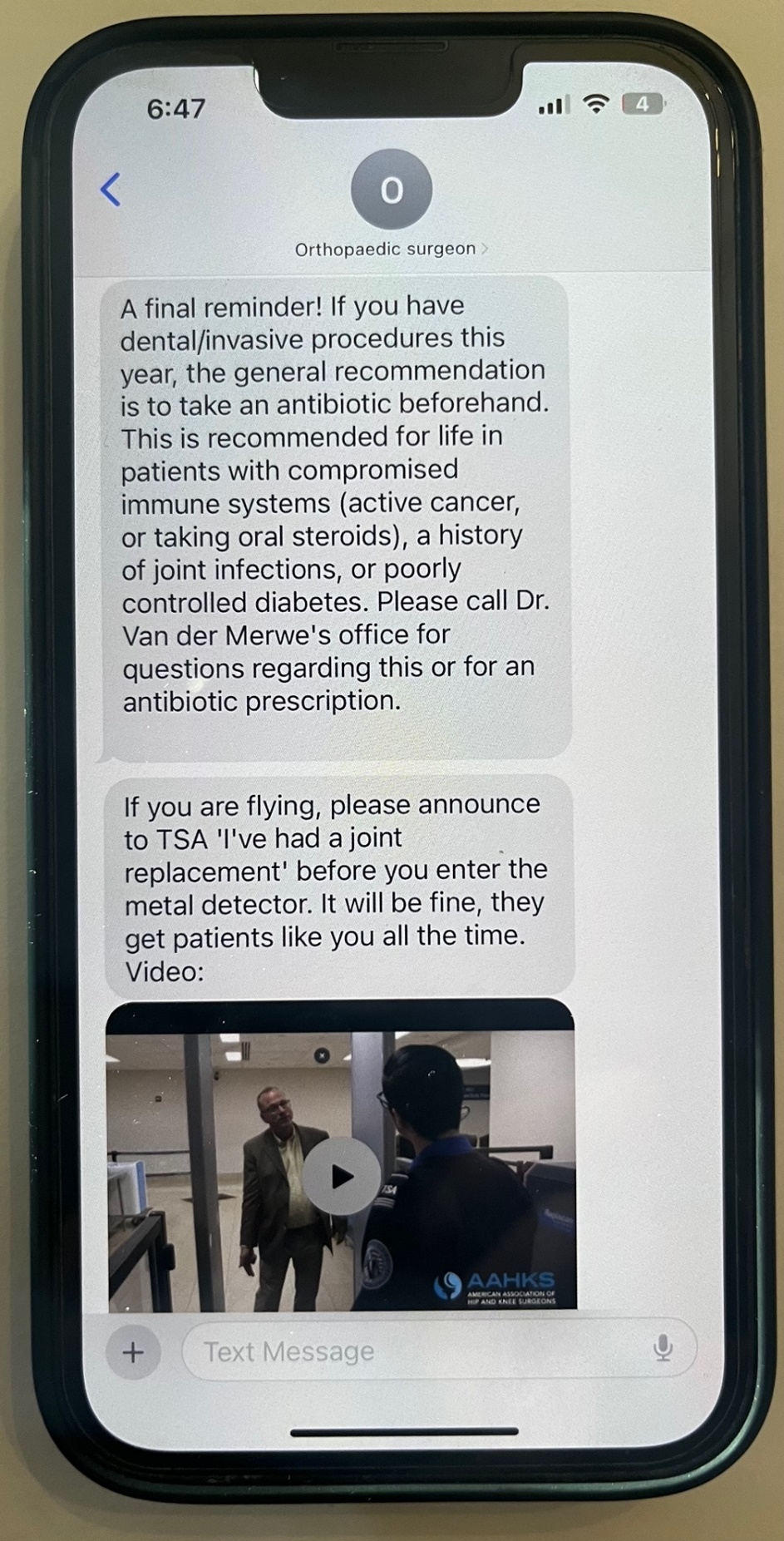
*

Supplement: Supplementary file 2 — Supplementary Material 2. Patient satisfaction questionnaire. [file 42836_2024_265_MOESM2_ESM.docx]
